# Associations between Manual Abilities, Gross Motor Function, Epilepsy, and Mental Capacity in Children with Cerebral Palsy

**Published:** 2014

**Authors:** Ewa GAJEWSKA, Magdalena SOBIESKA, Włodzimierz SAMBORSKI

**Affiliations:** 1 Chair for Physiotherapy, Department,, Rheumatology and Rehabilitation University of Medical Sciences, Poznań, 28 Czerwca 1956r. 135/147 61-545, Poznań, Poland

**Keywords:** Cerebral palsy, Motor function, Epilepsy, Mental retardation

## Abstract

**Objective:**

This study aimed to evaluate gross motor function and hand function in children with cerebral palsy to explore their association with epilepsy and mental capacity.

**Material & Methods:**

The research investigating the association between gross and fine motor function and the presence of epilepsy and/or mental impairment was conducted on a group of 83 children (45 girls, 38 boys). Among them, 41 were diagnosed with quadriplegia, 14 hemiplegia, 18 diplegia, 7 mixed form, and 3 athetosis. A neurologist assessed each child in terms of possible epilepsy and confirmed diagnosis in 35 children. A psychologist assessed the mental level (according to Wechsler) and found 13 children within intellectual norm, 3 children with mild mental impairment, 18 with moderate, 27 with severe, and 22 with profound. Children were then classified based on Gross Motor Function Classification System and Manual Ability Classification Scale.

**Results:**

The gross motor function and manual performance were analysed in relation to mental impairment and the presence of epilepsy. Epilepsy was found to disturb conscious motor functions, but also higher degree of mental impairment was observed in children with epilepsy.

**Conclusion:**

The occurrence of epilepsy in children with cerebral palsy is associated with worse manual function. The occurrence of epilepsy is associated with limitations in conscious motor functions. There is an association between epilepsy in children with cerebral palsy and the degree of mental impairment.

The occurrence of epilepsy, mainly in children with hemiplegia and diplegia is associated with worse mental capacities.

## Introduction

Functional problems associated with the motor impairments of cerebral palsy are complex and should be identified and addressed by doctors, speech therapists, physiotherapists, psychologists, and other essential specialists. This is due to the fact that in addition to the motor system impairments, such as paresis, involuntary movements, disorders of the coherence of movements (according to definition of the cerebral palsy), a number of disorders with various degrees of intensity are found, including epilepsy, disturbances of sensation, disorders of perception and communication, cognitive disorders, behaviour disorders and secondary musculoskeletal problems (1).

Epilepsy is listed among many associated problems in children with cerebral palsy. Its presence is connected with the increasing risk of cognitive problems, and it can cause larger problems with the care of a patient. 

Epilepsy occurs in 25-45% of children with cerebral palsy (2). According to Wallace, seizures are mainly of the symptomatic nature. If children with cerebral palsy have difficulties in learning at the same time, the risk of also having epilepsy increases up to 71% (3). 

It is estimated that among children with cerebral palsy only 10-32% will develop epilepsy, among children with mental impairment only 10-29%, and co-occurrence of cerebral palsy and the impairment is associated with 50% risk of epilepsy (1). In Wallace’s opinion, epilepsy is more frequent in some forms of cerebral palsy, e.g. it accompanies hemiplegia in 33-50%, where focal seizures dominate (69-73%), whereas according to Reid et al., it occurs in 24% (3-5). 

Epilepsy appears less frequently in spastic diplegia (approximately 16-27%) with the majority showing focal seizures (24%) and secondarily generalized seizures (50%) (4). Epilepsy appears in tetraplegia in 50-94% with the explicit dominance of West Syndrome (27%) and focal symptoms (22%), and with secondary generalisation (50%).

Cerebral palsy describes a group of permanent disorders in motor and postureal development, causing limitation of activities, which are attributed to non-progressive disturbances that occur during brain development of a fetus or an infant. 

Disturbances of skin sensation, disorders of perception and communication, cognitive disorders, behavior disorders, and secondary musculoskeletal problems frequently accompany motor disorders in cerebral palsy (6).

Sub-classifications of cerebral palsy are as follows:

spastic quadriplegia (spasticity involving about equal involvement all four limbs), spastic hemiplegia (spasticity of the arm and leg on one side), spastic diplegia (spasticity of the lower limbs more affected than the upper), athetoid, ataxic, and mixed cerebral palsy (manifestation of both spastic and extrapyramidal types) (6).

The aim of the present study was to evaluate gross motor function and hand function in children with cerebral palsy to explore their association with epilepsy and mental capacity.

## Material & Methods

This research was conducted in a special school and in Child and Youth Neurology Center in Poznań, Poland during 2006-2009.

Eighty-three consecutive patients of the outpatients neurologist office with diagnosis of cerebral palsy were enrolled. Forty-five (54.2%) patients were girls and 38 (45.8%) were boys, and their age was 12.2±4.2 years. In the investigated group of children, 41 (49.39%) were diagnosed with quadriplegia (A), 14 (16.86%) with hemiplegia (B), 18 (21.68%) with diplegia (C), 7 (8.43%) with mixed form (D), and 3 (3.61%) with athetosis (E). Epilepsy was diagnosed in 18 children with quadriplegia, 8 with hemiplegia, 3 with diplegia, and 6 with mixed form of cerebral palsy. 

Regarding epilepsy, the diagnosis made by a neurologist and placed in the patient’s chart, was taken into account, such that only the presence or absence of epilepsy was noted, without dividing it into subtypes. A psychologist who assessed children, ascribed them to appropriate degree of mental impairment. Thirteen children were classified as normal intellectual capacity, three children had mild impairment (IQ 69-55 according to Wechsler), 18 had moderate impairment (IQ 54-40 according to Wechsler), 27 had severe impairment (IQ 39-25 according to Wechsler), and 22 had profound impairment (IQ below 25 points according to Wechsler). 

The exact data are presented in Table 1. After evaluating the level of gross motor function according to Gross Motor Function Classification System (GMFCS) (7), the following distribution was observed: Level I= 13 children, Level II= 11, Level III=10, Level IV= 22, and Level V= 27. 

The entire group was also classified according to the Manual Ability Classification System (MACS) (8). The manual function in this group is presented as follows: 

Level I= 15, Level II= 9, Level III= 21, Level IV= 16, and Level V= 22. The Data are presented in Table 2. 

It was investigated whether mental impairment and motor function depended on the type of cerebral palsy and whether the presence of epilepsy influenced the motor function and/or mental impairment. 

All children’s Parents gave their informed consent for the study.

The results were undergone a statistical analysis using Statistica 10.0 program. Logistic regression was applied to assess the influence of epilepsy on GMFCS and MACS levels. 

## Results

**Fig 1:**
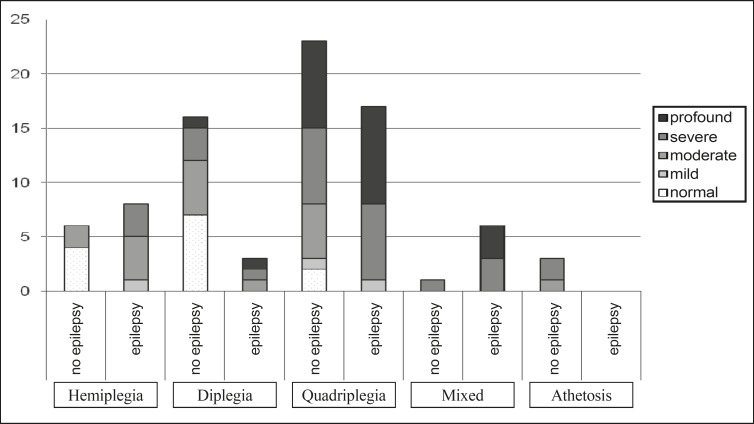
Number of children classified based on the type of cerebral palsy, mental impairment, and presence or not of epilepsy. The differences were statistically significant at p=0.035

Based on analysis of variance between non-parametric variables (Kruskal-Wallis test), it was checked whether mental impairment depended on the type of cerebral palsy. Severe and profound mental impairment was found to be significantly more frequent (p=0.002) in patients with tetraplegia, followed by patients with diplegia and hemiplegia. As far as the level of the mental impairment, the type of cerebral palsy, and the occurrence of epilepsy were considered, the occurrence of epilepsy in diplegia and hemiplegia was associated with higher level of the mental impairment, while for the mixed forms and quadriplegia, the occurrence of epilepsy did not show any difference with generally lower mental abilities. The difference was statistically significant (p=0.035) (Fig. 1). 

General motor performance was affected by the presence of epilepsy (p<0.01, chi2=12.573, odds ratio=1.01). 

Studying manual function (MACS) in relation to the occurrence of epilepsy, for all children taken together, a statistically significant difference was observed at p=0.035. In children with epilepsy, MACS was on a higher level (i.e., hand function was poorer). The differences among particular types of cerebral palsy were not significant, though the tendency for worse manual function in presence of epilepsy was always present. The presence of epilepsy statistically significantly influenced the MACS level (p<0.01, chi2=12.573, odds ratio=1.09). 

**Figure 2:**
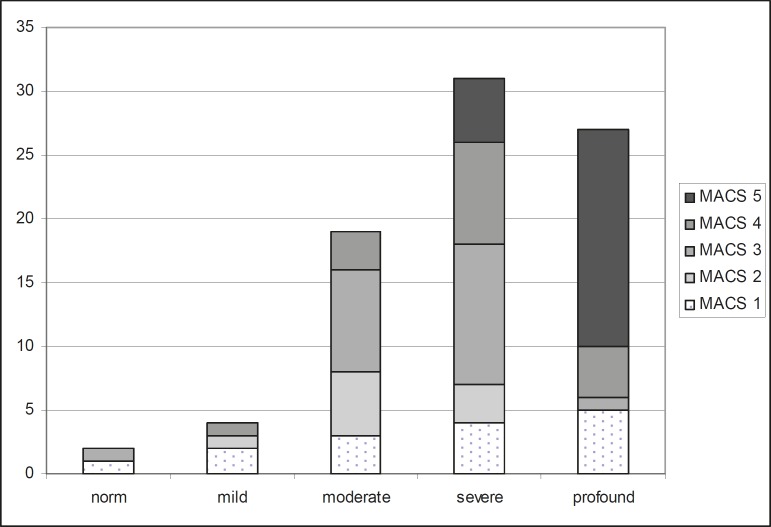
Number of children classified according to mental impairment (Wechsler) and Manual Ability Classification System (MACS) levels

The relation between MACS and mental impairment is shown in Fig 2. Children with profound mental impairment showed the most severe problems with manual function, while children with mild mental impairment had the mildest problems (p=0.000).

The highest MACS levels (i.e., the worst performance) were observed in children with quadriplegia, diplegia, and with mixed form of cerebral palsy, whereas children with hemiplegia showed the best manual performance (p<0.001).

There was no statistically significant correlation between manual function measured with the MACS scale and the level of motor abilities measured with GMFCS. 

However, there was a statistically significant correlation between mental impairment and GMFCS (p=0.000).

No child classified at GMFCS Level I showed profound mental impairment, whereas several children classified as Level IV or V showed deep mental impairment.

## Discussion

The number of children with motor disability in Poland is estimated at about 40,000-50,000, and more than a half of them (up to 25,000) have cerebral palsy and may vary between 1.5 and 3.0 per 1000 live births. It is relatively easy to exclude this disease if the physiological pattern of a child’s motor development is normal (9-12). It should be clearly pointed out, however, that the function of each hand and their cooperation is very individual in the case of children with cerebral palsy and it should be scored apart from the general assessment of the entire motor development. The perspective is necessary to plan the process of physical rehabilitation properly. It is well known that the normal development of manual function is connected with the development of visualmotor coordination, which is in turn related to the development of the remaining psychomotor spheres. There are many scales describing hand function, such as the questionnaire prepared by Arnold et al. or the Hellbrüge scale, both intended for children with cerebral palsy but without any intellectual disability (13, 14). Basing on broad researches and many years of study on children suffering from different types of cerebral palsy, a group of doctors and therapists designed MACS as system of manual ability classification (8) and demonstrated its effectiveness. Similarly to the Swedish group, some British authors believe that the MACS is a useful method of assessing the manual development of children with cerebral palsy in clinical practice as long as it is done by specialists (8, 15).

**Table 1:** The Level of the Mental Impairment and Occurrence of Epilepsy Depending On the Type of Cerebral Palsy (n=83)

**Cerebral palsy**	**Occurrence of epilepsy**	**Mental impairment**			
		**IQ** **69-55**	**IQ** **54-40**	**IQ** **39-25**	**IQ** **below 25**
**Quadriplegia, n=41**	18	2	6	14	17
**Hemiplegia, n=14**	8	1	6	3	-
**Diplegia, n=18**	3	-	5	4	2
**Mixed form, n=7**	6	-	-	4	3
**Athetosis, n=3**	-	-	1	2	-

**Table 2:** The Level of Manual Ability Classification System (MACS) and Gross Motor Function Classification System (GMFCS) Depending On the Type of Cerebral Palsy

**MACS levels**	**Quadriplegia n=41**	**h** **emiplegia** **n=14**	**d** **iplegia** **n=18**	**m** **ixed form n=7**	**a** **thetosis** **n=3**	**Total** **n=83**
**I**	1	6	8	0	0	15
**II**	5	1	1	2	0	9
**III**	7	6	6	1	1	21
**IV**	8	1	3	3	1	16
**V**	20	0	0	1	1	22
**GMFCS levels**						
**I**	0	7	6	0	0	13
**II**	3	3	4	1	0	11
**III**	1	1	4	3	1	10
**IV**	15	1	4	2	0	22
**V**	22	2	0	1	2	27

It has been shown in the current study that decrease of manual ability was associated mainly with those types of cerebral palsy, in which the cooperation between the two hemispheres of the brain was impaired. The presence of damage only on one side of the body seemed to have less influence on manual abilities. Carnahan, et al. who studied 365 children with cerebral palsy discovered that patients with hemiplegia presented a lower level of manual performance than the level of general development, it was an opposite relation in case of diplegia, whereas children with dyskinetic cerebral palsy presented the deepest impairment of the general and manual development (15). The present data showed no clear evidence of correlation between any type of cerebral palsy and manual function. 

In Bax’s research, 27% of hemiplegia cases were caused by stroke and 34% by asymmetrical periventricular leukomalacia, however, they could not be distinguished clinically. These patients started walking by themselves similar to their peers, but their hand function was much more disturbed. Significant differences appeared in spastic quadriplegia in comparison with spastic diplegia. These children mostly presented severe motor impairment (the levels IV and V of GMFCS), and very limited hand function (16). Similar to the data mentioned above, the present research showed that in patients with quadriplegia, the general level of gross motor function (GMFCS) was definitely on a lower level than in the patients with diplegia or hemiplegia. 

Many people with cerebral palsy have other problems, such as epilepsy or mental impairment, which hamper their functioning in everyday life; sometimes these problems may limit functioning to a higher degree than motor impairment. The impairments might be the result of the same or similar pathophysiological processes, which had led to disorders of motor activity. 

Karen, et al. studied the relation between the occurrence of epilepsy and the type of cerebral palsy. The research showed that epilepsy was associated mainly with the diagnosis of quadriplegia, then with the mixed form of cerebral palsy and hemiplegia (17). Odding suggested that epilepsy could be diagnosed in 20-40% of patients, with both hemiplegia and quadriplegia (18). Sugiura and co-authors claimed that prognosis of epilepsy was not related to the type of cerebral palsy; however, they showed that in the spastic children with epilepsy, a larger number of dysfunctions related to intellectual development might be noticed (19). As a result of the present study, it might be presumed that higher level of mental impairment was found in children with cerebral palsy who also had epilepsy. Considering the level of impairment, the type of cerebral palsy, and the occurrence of epilepsy it could be concluded that the presence of epilepsy significantly impaired mental capacities in children with hemiplegia and diplegia. In children with quadriplegia whose mental impairment was the deepest, epilepsy did not cause any further deterioration. 

McLellan believes that problems of behavior in children with cerebral palsy occurred more frequently in those who also had epilepsy and could be regarded as a measure of the size of neurological injury. The mental impairment occurred more frequently in patients with epilepsy than in patients with chronic diseases, in whom the injury of the central nervous system had not been diagnosed. According to the author, epilepsy might be an additional risk factor for the intellectual impairment in the group of children with cerebral palsy (20). Kulak and Sobaniec compared children with quadriplegia and diplegia and showed that mental impairment and epilepsy occurred more frequently in the group with quadriplegia (21). The present research showed that in the group of children with quadriplegia, the degree of the mental impairment was the most severe, and it was less severe in children with diplegia or hemiplegia. The issue of mental impairment occurring in children with cerebral palsy was also discussed by Rossman and Ashwal (22). They claimed that there were certain connections between the type of cerebral palsy and various cognitive impairments and that more severe mental impairment occurred in children with spastic quadriplegia than in children with hemiplegia. Motor disorders occurring in children with spastic cerebral palsy predispose them to a severe cognitive impairment, unlike children with dyskinetic type. They also revealed that there was a strong relation between the degree of the mental impairment in children with cerebral palsy and the occurrence of epilepsy, abnormal EEG, or abnormal neuroimaging study.

The present research did not show a significant influence of epilepsy on further impairment of general motor skills, whereas its presence was associated with lower manual ability of the studied patients. It is, however, difficult to relate to any previous researches since none of them investigated manual function apart from general motor function, showing only an unequivocal relation between the motor function and the type of cerebral palsy or the occurrence of epilepsy.

Obviously, every single child requires an individual approach, but parallel estimation of motor function disturbances and psychomotor development ought to become the issue of an interdisciplinary discussion of specialists dealing with children with cerebral palsy. 

Assessment by GMFCS and MACS cannot be used interchangeably in children with cerebral palsy, because both gross motor and manual abilities have to be described separately.

The manual function seems to be closely related to cognitive capacities and volitional control of movement.

There is often a big difference between the maximum ability (what many people call ”capacity”) and the spontaneous activity (called ”performance”), which is between what the child would be able to do and what he or she really does. The MACS assesses the latter factor and helps to form an opinion on how the child deals with everyday activities (8). MACS measures the conscious use of upper limbs rather than just the capacity, and a closer dependence on mental impairment and epilepsy was observed for manual function than general motor performance. These are the factors, which may considerably influence self-reliance of children with cerebral palsy in everyday life, and it is important to include some information on the above mentioned factors in the MACS assessment. It may be stated that mental impairment and epilepsy are the factors, which may significantly influence the self-reliance of children with cerebral palsy, and it would be important to include some information on the above factors in the MACS assessment.

**In conclusion**

1. The occurrence of epilepsy in children with cerebral palsy is associated with worse manual function.

2. The occurrence of epilepsy is associated with limitations in conscious motor functions.

3. There is an association between epilepsy in children with cerebral palsy and the degree of mental impairment. The occurrence of epilepsy, mainly in children with hemiplegia and diplegia is associated with worse mental capacities.

## Author contribution

Dr Ewa Gajewska was involved in selection of the group under study, physiotherapeutic assessment, obtaining data from medical history, writing the paper draft, and literature discussion; Dr Magdalena Sobieska performed statistical analysis, literature discussion, and paper discussion; and Professor Włodzimierz Samborski was the supervisor of the study, and performed discussion of the results and revised the paper.

## Conflict of interest

Authors declare no conflict of interest.

## Ethical approval

The study was conducted under a research Grant from the Ministry of Science and Higher Education KBN N N404269639.
